# The Cocaine Habit

**Published:** 1885-12

**Authors:** 


### The Cocaine Habit.

Louis Bauer, M. D., M. R. C. S., England, Professor of Surgery in the St. Louis College of Physicians, made a report to the Mississippi Valley Medical Society, at its meeting in September last, of a case of the cocaine habit in a man suffering from chronic alcoholism.

On the 9th of January last, he became so badly wrecked from his drinking, that he appealed to a physician for relief. The physician used cocaine hypodermically. Unfortunately, he dropped the name of the drug, which the patient caught up, and procured it for his own use. He used it hypodermically, beginning with one-fifth of a grain, increasing it to ten grains at a dose, and very soon found that he must use the drug continuously. The doctor reviewed the physiological action of the drug, so far as it is known, especially as it showed itself on this patient. He noticed that patient acquired a decided aversion for alcoholic liquors; that the cocaine habit was most deplorable for a man of his intellect and social station of life, but thought that the use of cocaine had injured him less than excessive alcoholism. He has observed none of those permanent organic disturbances which alcohol produces. By comparison, therefore, the former is preferable to the latter.

